# A Spring Compensation Method for a Low-Cost Biped Robot Based on Whole Body Control

**DOI:** 10.3390/biomimetics8010126

**Published:** 2023-03-21

**Authors:** Zhen Wang, Lei Kou, Wende Ke, Yuhan Chen, Yan Bai, Qingfeng Li, Dongxin Lu

**Affiliations:** 1 Department of Mechanical and Energy Engineering, Southern University of Science and Technology, Shenzhen 518055, China; 2 Institute of Oceanographic Instrumentation, Qilu University of Technology (Shandong Academy of Sciences), Qingdao 266075, China; 3 Health Management System Engineering Center, School of Public Health, Hangzhou Normal University, Hangzhou 311121, China

**Keywords:** spring compensation, linear quadratic regulator, whole body control, spring clearance compensation

## Abstract

At present, the research and application of biped robots is more and more popular. The popularity of biped robots can be better promoted by improving the motion performance of low-cost biped robots. In this paper, the method of the Linear Quadratic Regulator (LQR) is used to track a robot’s center of mass (COM). At the same time, the whole-body-control method and value function generated in the process of tracking COM are used to construct the quadratic programming (QP) model of a biped robot. Through the above method, the torque feedforward of the robot is obtained in the Drake simulation platform. The torque feedforward information of the robot is transformed into position feedforward information by spring compensation. In this paper, open loop control and spring compensation are used, respectively, to make the robot perform simple actions. Generally, after the compensation method of spring compensation is adopted, the roll angle and pitch angle of the upper body of the robot are closer to 0 after the robot performs an action. However, as the selected motion can introduce more forward and lateral motions, the robot needs more spring clearance compensation to improve performance. For improving the motion performance of a low-cost biped robot, the experimental results show that the spring compensation method is both reasonable and effective.

## 1. Introduction

In recent years, biped robots have attracted more and more attention in the field of robotics. The biped robot has a structure similar to the human body. People are more likely to want them to replace jobs in everyday environments. Currently, some entrepreneurs agree on the importance of robots and many of them have created robotics companies dedicated to the development of the robotics industry. For the commercialization of biped robots, reducing their cost is necessary. Low-cost biped robots are available in the current robotics market, but their use of low-cost motors, reducers, sensors, and other components may result in various errors in the robot itself. The low-cost and poor control accuracy of low-cost biped robots can be attributed to this reason. To facilitate better commercial development of such robots, it is necessary to implement methods to improve their control accuracy.

At present, there are many examples of low-cost bipedal robots such as France’s NAO, Leju’s Roban, UBTECH’s Qrobot, South Korea’s Darwin-OP and so on. For such low-cost robots, a model approach can be adopted to generate gait trajectories. For example, Kajita, the founder of the model generation method, proposed the linear inverted pendulum model [1]. This has also been successfully applied to ASIMO. Subsequent scholars in his experiment and other scholars also proposed the DCM model [2], SQD model [3,4,5], VIP model [6] and other models to generate robot trajectory on the basis of such models. Others such as Grizzle at the University of Michigan used nonlinear controls such as HZD to generate robot trajectories [7].

Based on these models, we can obtain the gait generation trajectory of a robot. However, in this process, the robot still has position error. For the work of error compensation, the current research has two kinds of solutions. One approach is based on dynamic analysis and the other uses machine learning-related algorithms. For the method based on dynamic analysis, some scholars have conducted relevant research. Qin proposed the Levenberg-Marquardt algorithm, and a variable parameter trajectory compensation model was designed in reference [8]. As a result, the accuracy of trajectory tracking was improved. Y. Xu proposed corresponding compensation rules for flexible robot wrist joints [9]. Wang proposed a feedforward compensation scheme based on the constant joint stiffness model in industrial robots [10]. Xue proposed a compensation algorithm for feedforward control based on the nonlinear friction and backlash characteristics of the robot [11]. P. Lischinsky implemented a model-based friction compensation scheme by using the dynamic friction model [12]. For the method based on machine learning correlation algorithm, some scholars have conducted relevant research. In recent years, some scholars adopted the improved whale optimization algorithm to reduce errors [13]. Other scholars used artificial neural networks to compensate position errors [14,15,16], and some scholars used similar methods of neural networks to reduce errors in robots, such as the fuzzy neural network (FNN) [17] and the dual regression adaptive domain adversarial neural network [18]. In this paper, the dynamic analysis method is adopted, and the joint driver of the robot is imagined as a spiral spring. By analogy with Hooke’s law, the law of elastic compensation is proposed. At the same time, this paper uses time-invariant LQR to design the COM tracking model. In this process, the optimal cost is used to generate the value function. The value function is also used as a part of constraint optimization of the whole-body motion control model. In this paper, the whole-body motion control model is solved by QP method. In this process, the torque feedforward of the biped robot is obtained. The torque feedforward is incorporated into the law of spring compensation. This method is used to improve the stiffness of hip joint of biped robot. In the follow-up experiments, spring compensation or spring clearance compensation was selected to improve the motion performance of the biped robot according to different motion trajectories. Finally, the compensation method makes the upper body of the low-cost biped robot more resistant to errors when doing squat leg, swing and other tasks. The primary contribution of this paper is the implementation of spring compensation through whole-body motion control, which has significant implications for the widespread use of biped robots.

The Roban robot, provided by Leju Company of China, was used in this paper. With a total of 12 degrees of freedom in its legs, it can achieve any pose in space. The second chapter describes the modeling process of tracking COM control, the third chapter describes the specific method of robot spring compensation, the fourth chapter describes the robot compensation experiment, and the fifth chapter gives the conclusion.

## 2. Problem Formulation and Assumptions

Low-cost biped robots are generally characterized by a low precision of joint control. This kind of robot is prone to large errors when it uses open-loop control to deal with simple tasks. In order to compensate the joint accuracy, this paper proposes a spring compensation method based on whole-body motion control. The Roban robot used the proposed method to complete an experiment in which it performed simple tasks. Experimental results show that the method is both reasonable and effective.

## 3. Com Tracking Control

As the robot moves, the actual COM will change. Our main purpose in controlling the COM is to control the stability of the upper body of the robot. To simplify the control process, we assume a fixed position instead of the COM of robot. Given that Roban is heavier in the upper body, we considered placing the COM somewhere in the upper body. A linear-quadratic controller is used to track the fixed position.

The biped robot is fully actuated. In this case, we can set the state-space equation of its COM as follows:

(1) $$\begin{array}{c} \dot{x}=Ax+Bu\\ =\left[\begin{array}{cc} 0 & I\\ 0 & 0 \end{array}\right]x+\left[\begin{array}{c} 0\\ I \end{array}\right]u\\ y=Cx+Du\\ =\left[\begin{array}{cc} I & 0 \end{array}\right]x+aIu \end{array}$$

where $x={\left[x_{\mathrm{com}},y_{\mathrm{com}},z_{\mathrm{com}},{\dot{x}}_{\mathrm{com}},{\dot{y}}_{\mathrm{com}},{\dot{z}}_{\mathrm{com}}\right]}^{T}$, $u={\left[{\ddot{x}}_{\mathrm{com}},{\ddot{y}}_{\mathrm{com}},{\ddot{z}}_{\mathrm{com}}\right]}^{T}$, **a** is the gain coefficient tracking the acceleration of the COM, which we set to 0.1. **I** is the identity matrix of the third order.

We wanted to keep the upper body stable, so the robot could complete the desired COM trajectory. Here we introduce the cost function J. We designed a LQR [19].

(2) $$J=\int_{0}^{\infty }\left(x^{T}Qx+u^{T}\mathrm{Ru}\right)\mathrm{dt}$$

We set $Q=10\left[\begin{array}{cc} I & 0\\ 0 & I \end{array}\right]$ and R = 0.1 **I**, and the matrix gain K as well as the quadratic cost term S can be obtained by solving J. The optimal cost function of the time-invariant linear system can be obtained by solving the Riccati equation.

(3) $$J^{*}\left(\bar{x}\right)={\bar{x}}^{T}S\bar{x}$$

The magnitude and direction of COM acceleration is influenced by the force of the joint actuator and the plantar friction. To control the input, we take the constraint minimization of the V function.

(4) $$V\left(\bar{x},\bar{u}\right)={\bar{y}}^{T}Q\bar{y}+{\frac{\partial J^{*}}{\partial \bar{x}}|}_{\bar{x}}\bar{x}$$

We deal with the above V function and put the y term of Equation (1) into the V function. The final form of the function expression looks like follow:

(5) $$V\left(\bar{x},\bar{u}\right)={\bar{x}}^{T}Q\bar{x}+u^{T}R\bar{u}+{\frac{\partial J^{*}}{\partial \bar{x}}|}_{\bar{x}}^{}\dot{\bar{x}}$$

where $\bar{x}=x-x^{d}$, $\bar{u}=u-u^{d}$.

## 4. Spring Compensation Modeling

Low-cost bipedal robots generally use steering gear as their actuator. When the robot moves, the steering gear will produce errors. At this point we can imagine the steering gear as a coiled spring. According to Hooke’s law:

(6) F=KX

where F stands for the tensile force on the spring, K is the spring coefficient of the spring and X is the shape variable of the spring.

According to the analogy of the above Equation (6), the spring compensation formula in steering gear can be obtained as follows [20]:

(7) $$q_{des}=q+k\tau$$

The $q_{des}$ is the planned steering gear rotation position; q is the actual rotating position of the steering gear; k is the spring compensation coefficient; and τ is the driving torque of steering gear.

The steering gear of conventional low-cost foot robots cannot accept force control. Therefore, the torque is converted into position by spring compensation, and the position information is transmitted to the steering gear. This is also important for low-cost biped robots to complete planned movements.

In the motion of the robot, its $q_{\mathrm{des}}$ and q are very easy to obtain. The most critical variables in spring compensation modeling are spring compensation coefficient k and torque feed forward τ. In this paper, the whole-body-control method is adopted to solve the driving torque required by the robot. Next, the modeling process of whole-body-control is reviewed.

### 4.1. Floating Base Dynamics Function

Let us briefly review the Lagrangian form of rigid body dynamics for robots. The expression is as follows [21]:

(8) $$H\left(q\right)\ddot{q}+C\left(q,\dot{q}\right)=B\left(q,\dot{q}\right)\tau +\Phi {\left(q\right)}^{T}\lambda$$

where H(q) is the system inertia matrix, $C\left(q,\dot{q}\right)$. captures the gravitational and Coriolis terms, $B\left(q,\dot{q}\right)$ is the control input map, $\lambda ={\left[\begin{array}{ccc} \lambda_{1}^{T} & \ldots & \lambda_{N_{c}}^{T} \end{array}\right]}^{T}$ is a vector of ground-contact forces acting at $N_{c}$ contact points and $\Phi {\left(q\right)}^{T}$ transforms external forces into generalized forces.

The base of the biped robot is floating. In this paper, it is assumed that the claw of the robot is also the floating base of the robot. This will facilitate our analysis of the robot’s feet. Finally, the Equation (8) is rewritten into the dynamic form about the floating base. As follows:

(9) $$\begin{array}{c} H_{f}\ddot{q}+C_{f}=\Phi_{f}^{T}\lambda \\ H_{a}\ddot{q}+C_{a}=B_{a}\tau +\Phi_{a}^{T}\lambda , \end{array}$$

Through Equation (9), we adopt the direct calculation torque method, and we can obtain:

(10) $$\tau =B_{a}^{-1}\left[H_{a}\ddot{q}+C_{a}-\Phi_{a}^{T}\lambda \right]$$

We obtain the acceleration and contact force of the actuator of the robot. Substituting them into Equation (10), we finally get the driving torque of the robot in the process of spring compensation.

### 4.2. Frictional Constraints

**Figure 1:**
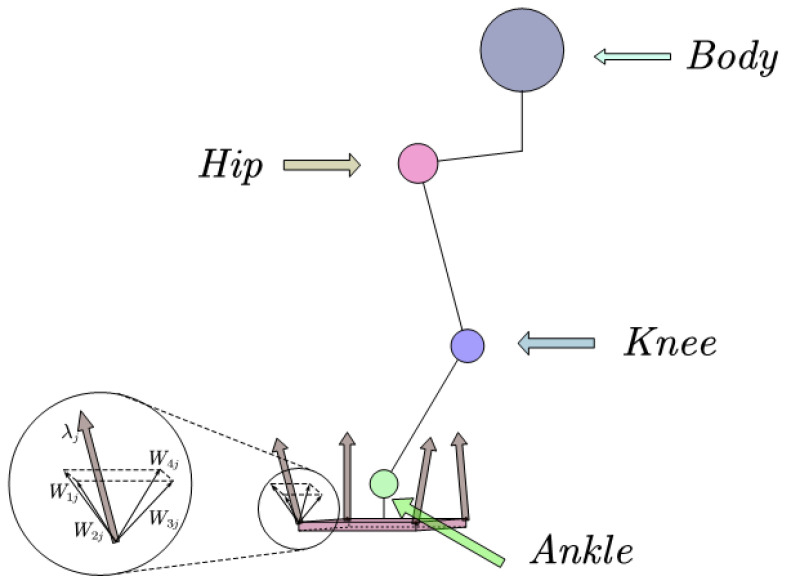
The simplified plantar of the robot.

The foot robot creates friction when it interacts with its environment. This has an impact on the overall motion of the robot. In order to make the robot control effect better, the friction force must be considered in the whole-body-control of the robot. The structure picture of a single leg of a simplified robot is given. A polyhedral approximation friction cone model is introduced in Figure 1 below.

According to the aspect ratio of the robot’s feet, Roban’s feet can be simplified into rectangles. We assume that there should be four contact points on each foot of Roban robot. Each contact point is at the top angle of its rectangle. In order to simplify the calculation process of the contact force, the friction cone model is replaced by the quadrilateral pyramid approximation. We can obtain [22,23]:

(11) $$\lambda_{j}=\overset{N_{d}}{\underset{i=1}{\sum }}\beta_{ij}w_{ij},\beta_{ij}\geq 0$$

In Equation (11), $N_{d}$ = 4. It represents the number of edges of the pyramid after the friction cone is simplified. $w_{ij}=n_{j}+\mu_{j}d_{ij}$, where $n_{j}$ is the normal phase vector of the contact surface; $d_{ij}$ is the vector of the ith parallel contact surface of the jth contact point; and $\mu_{j}$ is the Coulomb friction coefficient.

### 4.3. Calculation of Joint Torque

Through Equation (10) obtained by the direct torque method, we know that the driving torque can be obtained by using the joint acceleration of the actuator and the overall contact force of the biped robot. To obtain them, the whole-body-control method is used for calculation.

The approach can consider two broad categories of problems. One is the dynamics of the floating base of the upper body of the biped robot and the other is the interaction between the foot and the environment of the biped robot. The common method to solve this kind of problem is quadratic programming (QP). We next adopt the QP approach to deal with the above problems. Briefly review the establishment of the QP equation [21].

(12) $$\min_{\ddot{q},\beta ,\lambda ,\eta }V\left(\bar{x},\bar{u}\right)+\omega_{\ddot{q}}|{\left|{\ddot{q}}_{des}-\ddot{q}|\right|}^{2}\;+\varepsilon \underset{ij}{\sum }\beta_{ij}^{2}+\left||\eta \right|{|}^{2}$$

subject to:

(13) $$H_{f}\ddot{q}+C_{f}=\Phi_{f}^{T}\lambda$$

(14) $$J\ddot{q}+J\dot{q}=-\alpha J\dot{q}+\eta$$

(15) $$B_{a}^{-1}\left(H_{a}\ddot{q}+C_{a}-\Phi_{a}^{T}\lambda \right)\in \left[\tau_{\min},\tau_{\max}\right]$$

(16) $$\forall_{j=\left\{1\ldots N_{c}\right\}}\lambda_{j}=\overset{N_{d}}{\underset{i=1}{\sum }}\beta_{ij}v_{ij}$$

(17) $$\forall_{i,j}\beta_{ij}\geq 0$$

(18) $$\eta \in \left[\eta_{\min},\eta_{\max}\right]$$

The Equations (13) and (15) guarantee the rigid body dynamics limitation. The Equation (13) is a restriction on the dynamics of the floating base. The Equation (15) is a limit on the range of the driving torque. This prevents damage to the robot’s actuators. The purpose of Equation (14) is to limit the sliding of the biped robot on the ground. J is the Jacobian matrix of the robot. α is the gain coefficient of Equation (14). Equations (16) and (17) adopt the method of friction cone to limit the plantar contact force of the robot during movement. At present, there are roughly three methods to solve QP problem. The first is a solver based on active-set methods, such as qpOASES [24]. The second one is a solver based on interior-point methods, such as OOQP [25]. The third is the alternating direction method of multipliers (ADMM) solver, such as OSQP [26]. There are also nonlinear solvers for QP problems, such as ipopt. After comprehensive consideration, we adopted the OSQP solver to solve the QP problem established above.

### 4.4. The Spring Compensation

Through the above process, the actuator driving torque of the robot can be solved. According to Equation (7), we also need to obtain the spring compensation coefficient of each actuator of the robot. In order to obtain the spring compensation coefficient of the robot, we can use the online method or the offline method.

For the online method, we can use the simulated annealing algorithm to generate the spring compensation coefficient of the robot in real time. The simulated annealing algorithm is a random search algorithm inspired by the principle of metal annealing. The algorithm is based on a hill-climbing search and can search the global optimal solution in the solution space. In our experiments, we used a low-cost biped robot that had limited real-time processing power, which posed some constraints on our ability to obtain real-time solutions. If we were to use a slightly more complex trajectory in our experiments, we might encounter real-time issues in determining the spring compensation coefficient.

As we model the robot’s actuator as a coiled spring during spring compensation, we can apply the principles of Hooke’s law to determine that the spring coefficient remains constant under the following two conditions: that the temperature of the spring is constant and that the spring is not subjected to tension beyond its carrying capacity. By analogy, there is a situation where the actuator of the robot does not fail and does not bear the force for a protracted period of time, resulting in the temperature increase of the actuator. We can assume that the spring coefficient of the robot actuator in this case is constant. Then, the off-line spring coefficient can be obtained by measuring the motion of the robot in each frame. We give the steering gear joint angle obtained from the inverse solution of the robot motor action in the first frame. The robot will perform the action of the first frame. The rest of the frame does the same thing. When the robot’s action is stable, the encoder can measure the current joint angle of each steering gear. Finally, the spring compensation coefficient of each frame was calculated by the Equation (7). Generally, the spring compensation coefficient of each steering gear of the robot can be obtained by the least squares fitting method.

## 5. Results and Discussions

**Figure 2:**
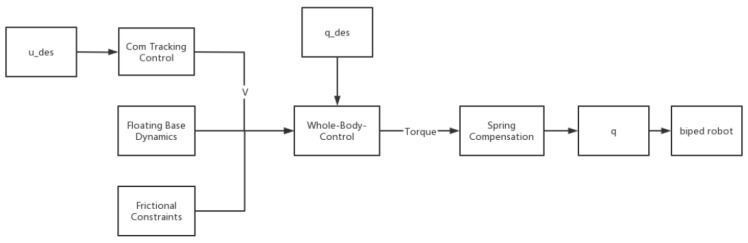
Control block diagram of spring compensation.

Through the previous chapter, we have drawn up the error compensation scheme of the robot. Next, we verify the rationality of the scheme through experiments. In the process of the robot squatting with its legs, the movement is stable. Such actions may not visually manifest the benefits of error compensation. Therefore, we designed the movement of the robot maintained on one leg, the movement of the robot squatting three times on one leg, and the movement of the robot swinging as the experimental movement of the robot. Furthermore, the experiment involved plotting the roll and pitch angle curves of the robot, which provided a visual representation of the effectiveness of error compensation. The compensation block diagram is depicted in Figure 2 below.

In order to complete the subsequent experiments, before the trajectory planning of the robot, we exported the model file of Roban SolidWorks into an URDF file. We can define the joint coordinates of the robot in the URDF file. The SolidWorks file of the Roban robot and the distribution of its steering gear are shown below.

**Figure 3:**
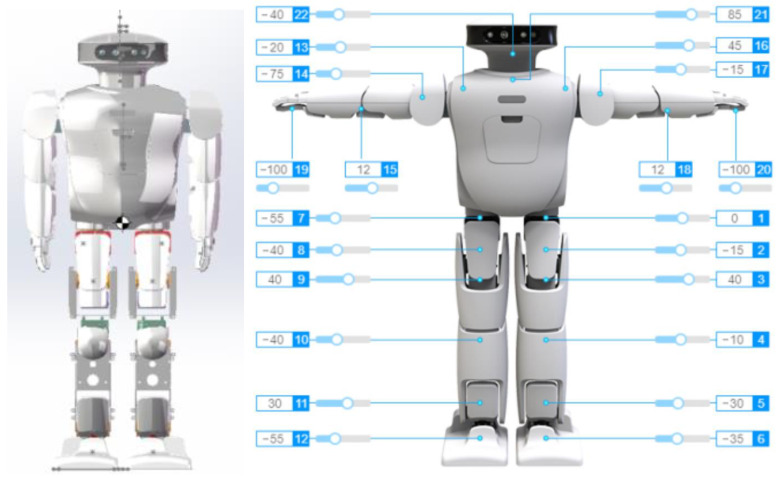
SolidWorks model of Roban and its steering gear distribution.

The Roban robot shown in Figure 3 is provided by Leju Company, Shenzhen, China. The figure on the right indicates the number of the steering gear and the zero drift of the robot. Roban has a depth camera and a corresponding vision algorithm. It can also complete interior mapping and navigation. At present, it is mainly used for efficient scientific research and artificial intelligence education. Specific parameters of Roban are shown in Table 1 below:

### 5.1. Trajectory

When we planned the trajectory, we needed to ensure that the kinematics of the robot could be inversely solved successfully. This process was carried out on the Drake simulation platform [27]. We first designed the trajectory of the robot’s three squats. We broke the action down into three stages. The first stage was the translation stage of the robot’s COM. The COM of the robot was shifted to the left. The second stage was the stage when the robot lifted its legs. The robot raised the right thigh. In the third stage, the robot completed a squat with a single leg. Each segment action was interpolated to obtain its position information. The one-leg maintenance could be considered the first two stages of the single-leg squat.

In order to test the rationality of the spring compensation method for robot global, we planned the experiment of the robot swinging with a single leg. When we completed the first phase of the COM shift, we made the feet of the robot move forward or move sideways.

According to the content of Chapter 3, in the above process we assumed that the position near the hip joint was the approximate robot COM. We added an inertial detection unit (JY901) at this location. In this way, it was convenient for us to obtain the information of roll and pitch angles of the robot.

**Figure 4:**
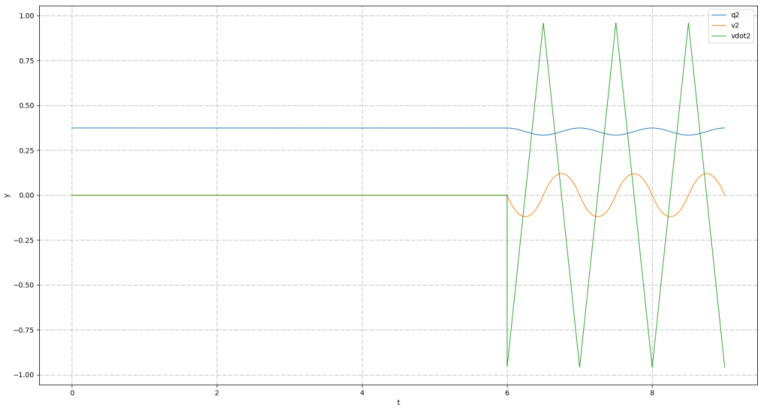
Planned COM motion trajectory diagram.

Figure 4 represented the planned COM trajectory of Roban in the Z direction. As can be seen from the picture, the robot completed three squats in the third stage.

We took a whole-body-control approach to compute the robot joint torque feed forward. To do that, we needed information about the velocity and acceleration of the robot. We used the interpolation method to obtain the robot motion trajectory. There were many discrete periods in each trajectory phase. In the whole trajectory planning stage, we obtained the velocity and acceleration information of the Roban robot in this trajectory by adopting Newton’s kinematic method, according to the following formula:

(19) $$v\left(t\right)=\underset{\Delta t\to 0}{\lim}\frac{x\left(t+\Delta t\right)-x\left(t\right)}{\Delta t}$$

(20) $$a\left(t\right)=\underset{\Delta t\to 0}{\lim}\frac{v\left(t+\Delta t\right)-v\left(t\right)}{\Delta t}$$

In interpolation, we chose a very small period dt. The advantage of this choice is that the instantaneous velocity and the instantaneous acceleration at that moment are approximately equal to the average velocity and the average acceleration over a period dt. Therefore, we can obtain the formula of the robot’s COM velocity and COM acceleration as follows:

(21) $$v\left(t\right)=\frac{x\left(t+dt\right)-x\left(t\right)}{dt}$$

(22) $$a\left(t\right)=\frac{v\left(t+dt\right)-v\left(t\right)}{dt}$$

Through the above method, the joint position information, velocity information and acceleration information of the Roban robot are stored as CSV files. We conducted simulation experiments and robot experiments through offline control.

### 5.2. Elastic Coefficient

In the error compensation model, the most crucial aspect is obtaining the spring coefficient of the robot joint. As different servos have varying errors, it is not feasible to dismantle the installed robot and measure its data to fit the spring coefficient using other methods like least squares. Doing so would introduce more installation errors. A robot is a system with high coupling. Obtaining multiple sets of different measurements directly from the robot would lead to inaccurate robot compensation.

We chose the trial-and-error method to calibrate the spring coefficient. Firstly, the data of the robot under standing was measured, and the reference spring compensation value was calculated by Equation (7). Based on this value, we only calibrated the spring compensation coefficients of the key steering gear. For example, the leg structure of the Roban robot was the standard 2-3-1 structure. For the roll angle direction, the NO. 2 steering gear of the left hip joint was the most affected joint. For the pitch angle direction, the NO. 3 steering gear of the left thigh was the most influential joint. We first calibrated the spring compensation coefficients of the four steering gears. The rationality of this method is verified by the experiment of a single leg robot which will maintain stability.

In the standing condition, we obtained the measurement data of the robot and calculate the spring compensation coefficient roughly. As shown in Table 2 below:

The key spring coefficients of NO. 2, 3, 8 and 9 steering gear in the above table were used to carry out the experiment of maintaining the stability of the single leg of the robot. The results were not satisfactory. We carried out a single direction of trial-and-error calibration for them. We can obtain spring compensation coefficient as shown in Table 3:

In order to obtain the effect of spring compensation visually, we first designed a control experiment. The control experiment allows the robot to maintain stable movement on a single leg only by means of position compensation. We move the COM of the robot by 5 cm during the shift.

**Figure 5:**
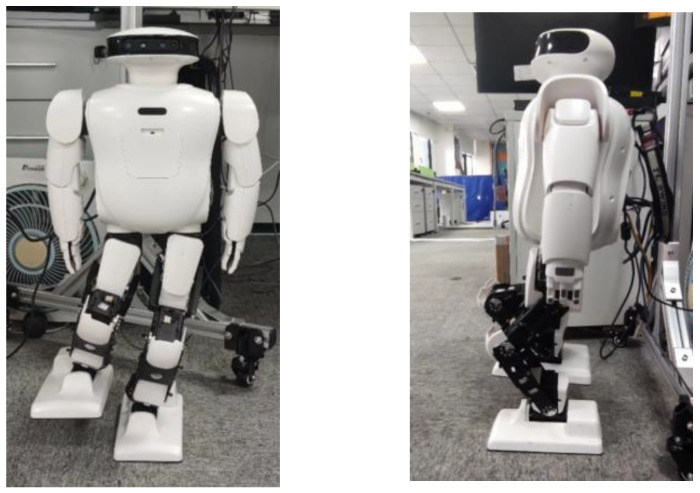
Roban stands on a single leg.

**Figure 6:**
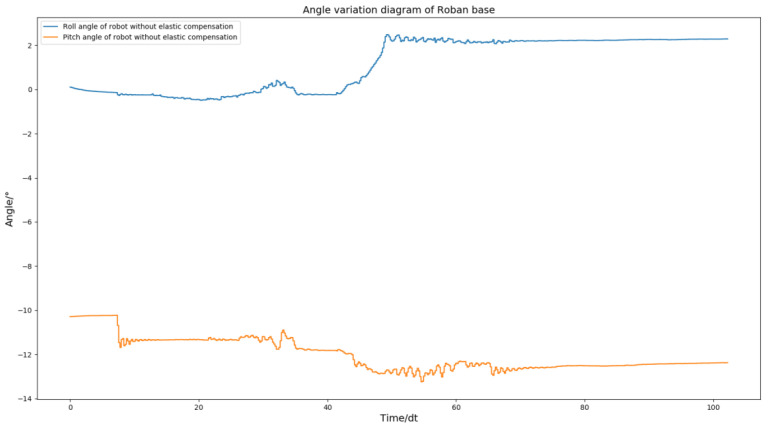
Roll angle and pitch angle change curve of Roban standing on a single leg under position control.

According to the Figure 5, it can be seen that the robot can complete the stable action of standing on a single leg when only using position control. However, there is a certain inclination in the hip joint. The curves of roll angle and pitch angle of the Roban robot were plotted during its movement, as shown in Figure 6.

From Figure 6, we can find that the roll angle and pitch angles of the robot after stabilization are shifted from 0 position by a lot. The robot’s plantar is planned to be parallel to the ground during movement. However, if there is an offset in the roll and pitch angles of the robot, it can cause the plantar to be non-parallel to the ground, leading to instability in the robot’s motion. In order to make the robot more stable, we adopted the spring compensation control method to perform the experiment of the same motion trajectory.

To ensure the execution of the spring compensation experiment, the corresponding action trajectory must be executed in the simulation to obtain the torque feed forward during the process. The kinetic library used in our study was Drake. Therefore, before doing the robot ontology experiment, we needed to import Roban’s URDF model into Drake. According to the contents of Section 3 and Section 4, we established the dynamic and kinematic models of the robot and developed a reliable spring compensation model. By using the built-in simulation software of Drake, we successfully executed the motion of the robot’s single leg to maintain stability.

**Figure 7:**
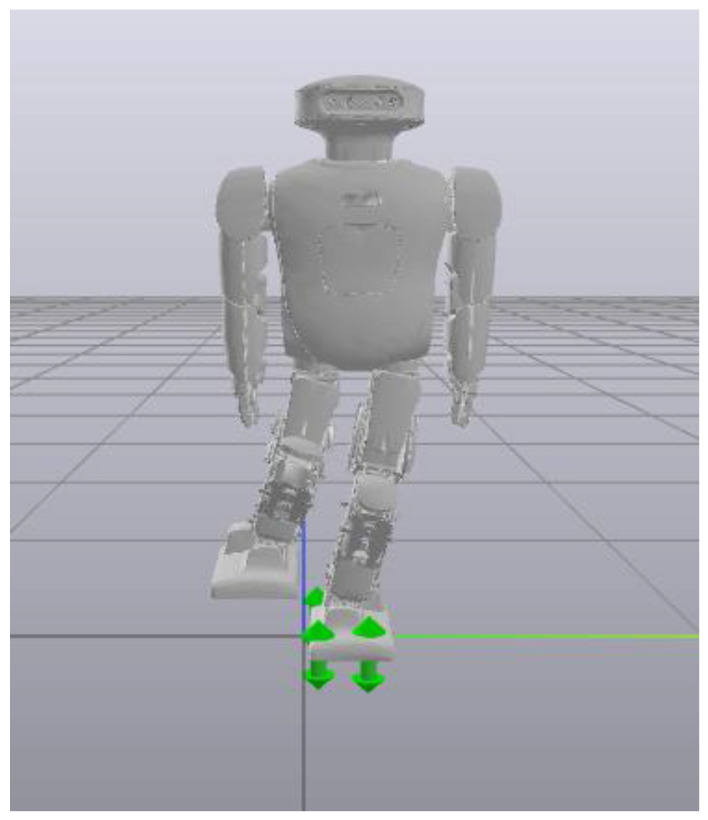
A Drake visualization of Roban standing on a single leg. The green symbols represent the plantar contact force of the robot.

According to Figure 7, it is shown that the robot could complete the one-legged standing action in the simulation environment. Through this simulation process, we were able to obtain the joint torque feed forward of the robot according to the whole-body motion control model. Torque feed forward was crucial for subsequent spring compensation.

**Figure 8:**
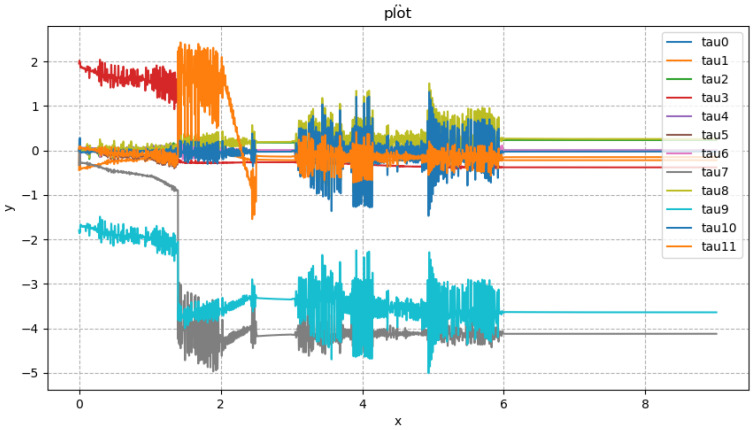
Torque feed forward for Roban standing on a single leg.

**Figure 9:**
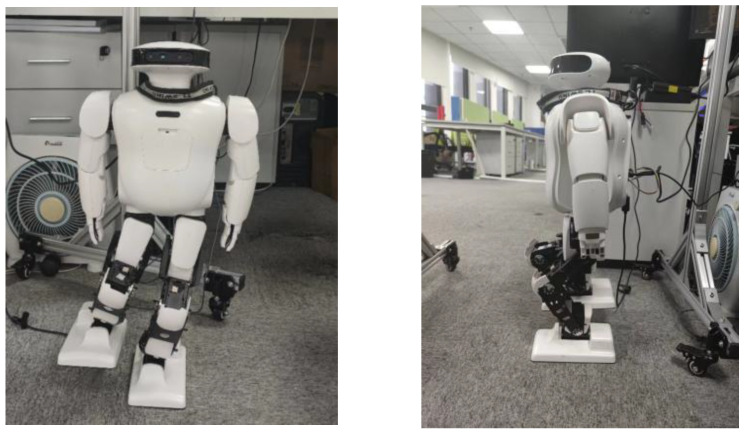
Roban standing on a single leg with spring compensation.

In Figure 8, the direction of x represents the planned time, and the direction of y represents the specific torque. The subsequent spring compensation experiments were carried out in Drake’s simulation software. This simulation phase was not repeated in the physical experiment. The compensation coefficient in Table 3 was used for the experiments. The CSV file containing the robot’s torque feed forward, which was obtained from the simulation, was used. The physical experiment was conducted to test the stability of the robot’s single leg. This experiment selected the planned trajectory consistent with the robot action shown in Figure 5. We chose to compensate the roll direction and the pitch direction of the robot, respectively. Figure 9 shows the stable state of the Roban robot in the experiment.

The above Figure 5 and Figure 9 demonstrate that the robot remains stable and does not fall when its COM moves 5 cm to the left, regardless of whether spring compensation is implemented or not. However, the effect of spring compensation on the roll angle of the robot is not easily discernible. Thus, we obtained the roll and pitch angle curves of the robot during the spring compensation experiment, and compared them with the curve in Figure 6.

**Figure 10:**
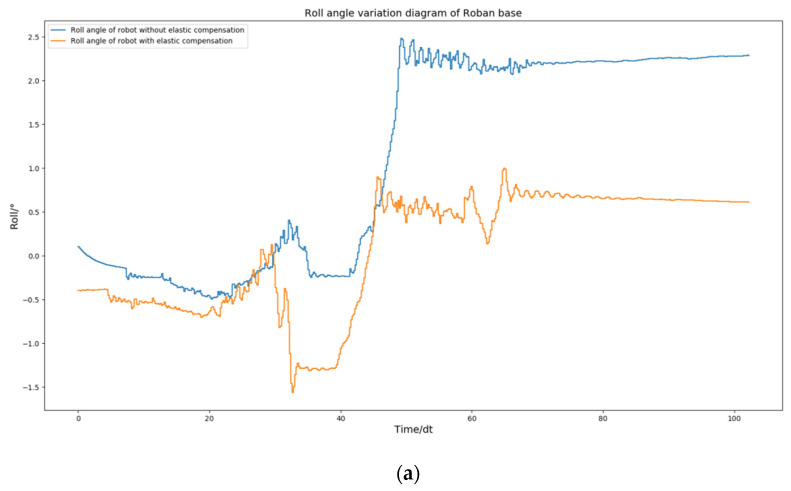

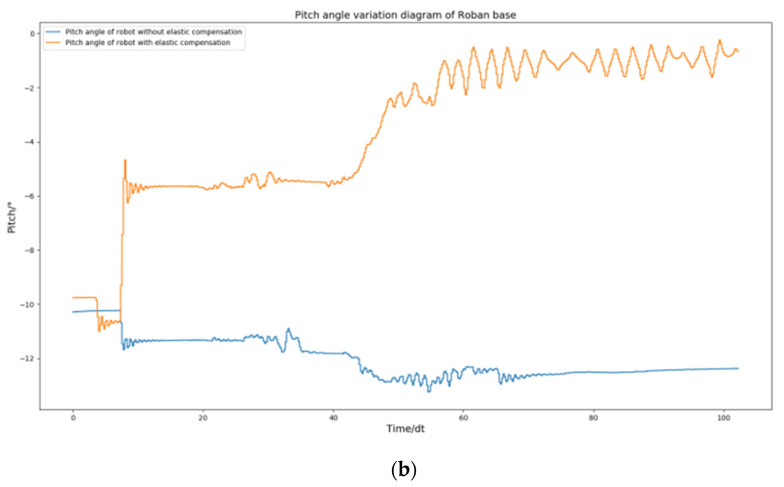
Roban stands on one leg. In this process, we chose to use or not use spring compensation control mode. Figure (**a**) is the comparison of the roll angle of the robot; Figure (**b**) is the comparison of robot pitch angle.

As shown in Figure 10, The robot’s roll angle was ultimately maintained near 0 by solely utilizing the spring compensation value of steering gear NO. 2. Similarly, the spring compensation value of NO. 3 alone was able to keep the robot’s pitch angle near 0. According to the experimental results, the method of spring compensation of the key steering gear is effective in dealing with the static motion of the robot.

### 5.3. Squat Experiment

We aim to increase the intelligence of robots, which requires them to perform dynamic actions and maintain stability. To test this, we had the robot perform three squats on a single leg to achieve a stable final state. This allowed us to further verify the effectiveness of the spring compensation.

We applied the squat motion trajectory, which was drawn up before the experiment, to the Roban robot. Firstly, we completed the physical squat action through position control.

**Figure 11:**
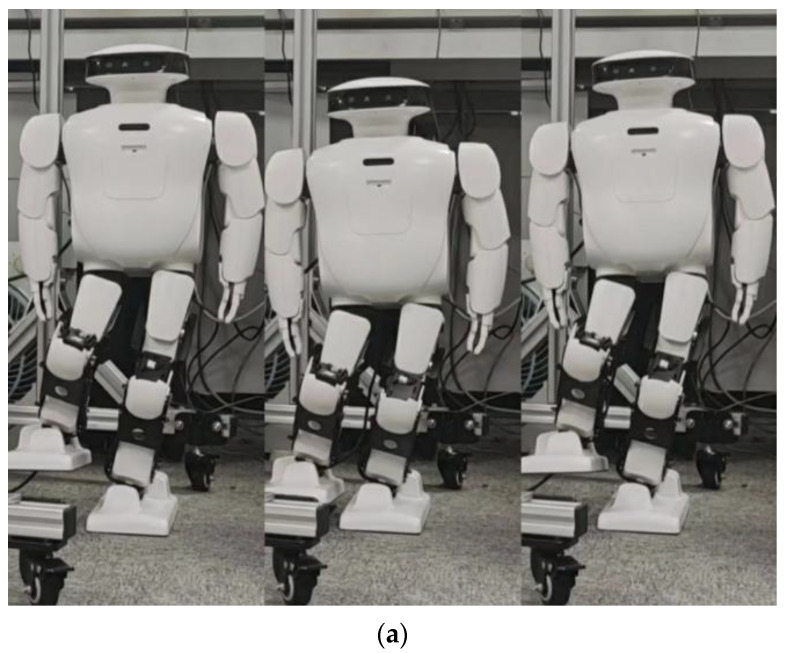

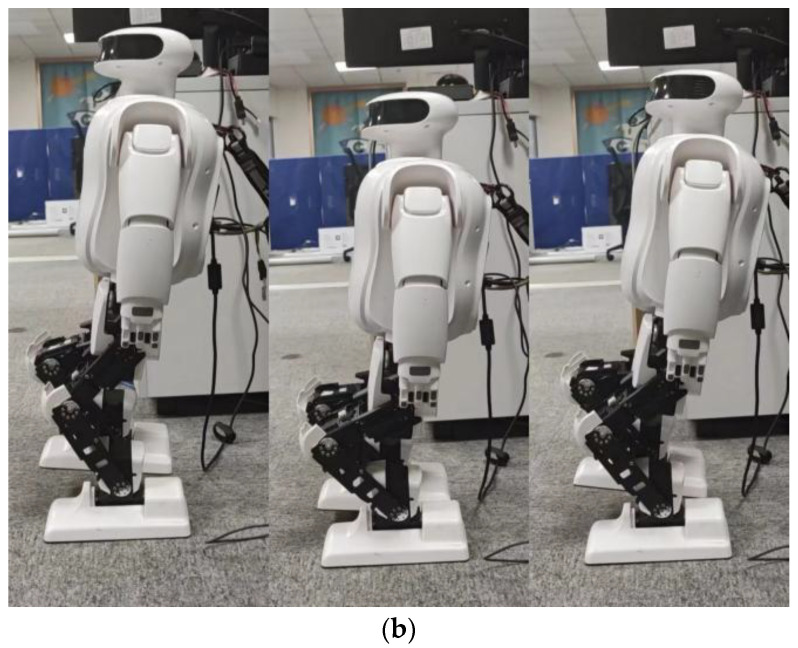
When Roban’s COM moves 5 cm, it completes three squats on a single leg. (**a**) A main view of the robot squatting process; (**b**) A side view of the robot squatting process.

Figure 11 shows a picture of the robot completing the squat when the COM is shifted by 5 cm. According to the picture, the hip joint of the robot has a large deviation during squatting. Even if there are errors, the robot can still complete the whole planned action. Next, we examine the change in roll angle and pitch angle of the robot in this process.

**Figure 12:**
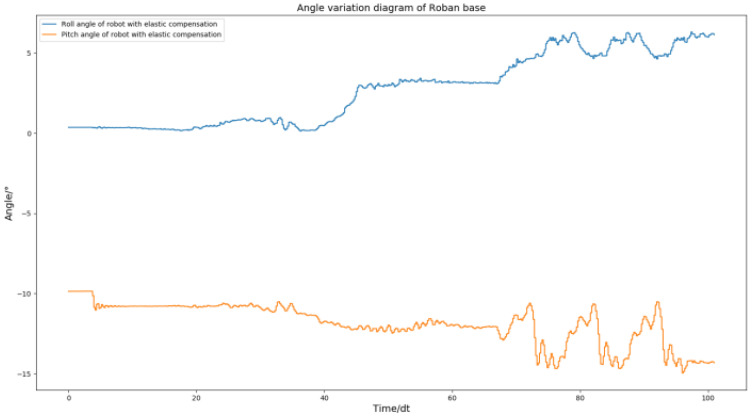
When Roban’s COM moves 5 cm, it completes three squats on a single leg. The changes of roll angle and pitch angle in this process.

**Figure 13:**
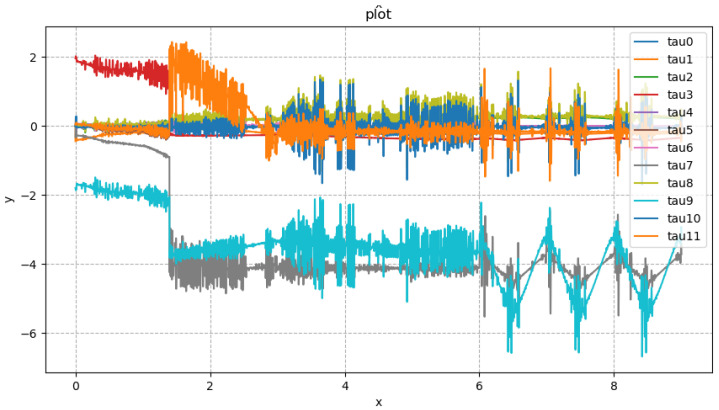
When Roban’s COM moves 5 cm, it completes three squats on a single leg. The torque feed forward diagram is required by Roban in this process.

According to Figure 12, the idea that the Roban robot has large error under pure position compensation is verified. In order to compensate for the error, we adopted the spring compensation method. In the Drake platform, the joint torque feed forward of the robot is obtained through the aforementioned simulation experiment as shown in Figure 13.

Next, we will use spring compensation for both the roll and pitch directions of the robot. The robot then completes the single-leg squat.

**Figure 14:**
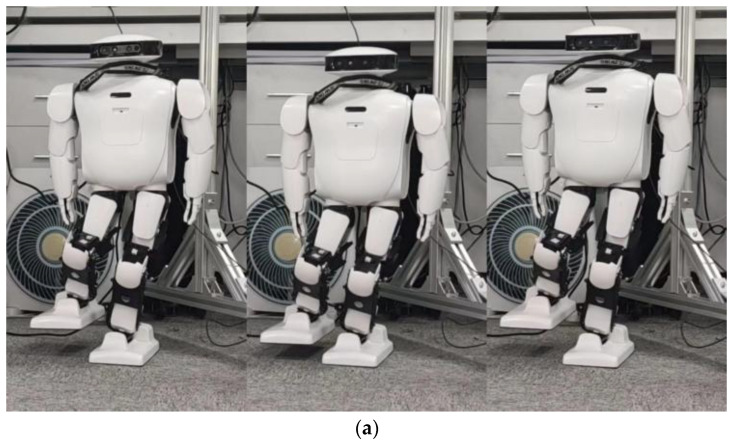

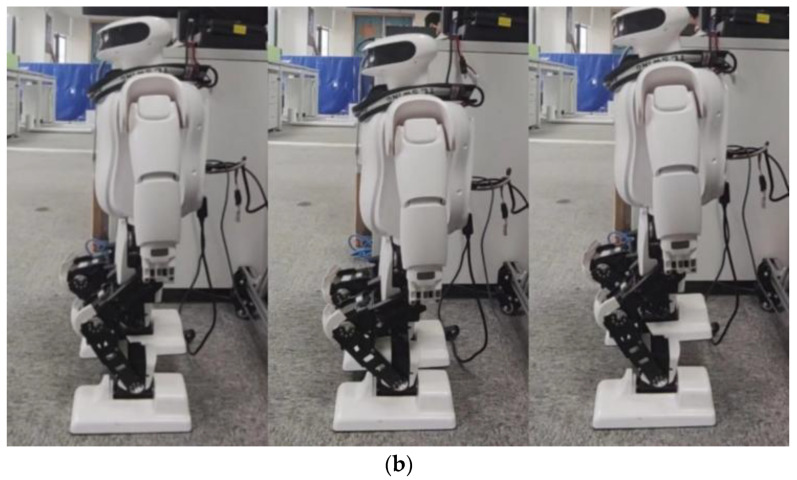
When Roban’s COM moves 5 cm, it completes three squats on a single leg with spring compensation. (**a**) A main view of the robot squatting process with spring compensation. (**b**) A side view of the robot squatting processwith spring compensation.

By comparing Figure 11 with Figure 14, the effect of spring compensation can be seen intuitively. Next, using the compensation curve of the robot, we compare it with results shown in Figure 12.

**Figure 15:**
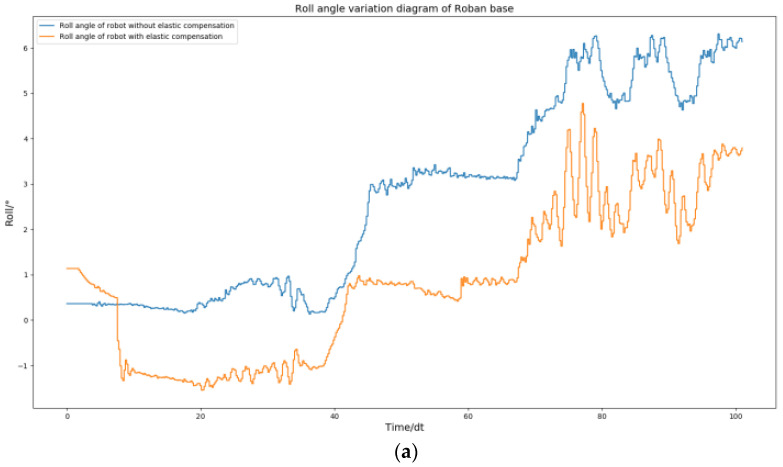

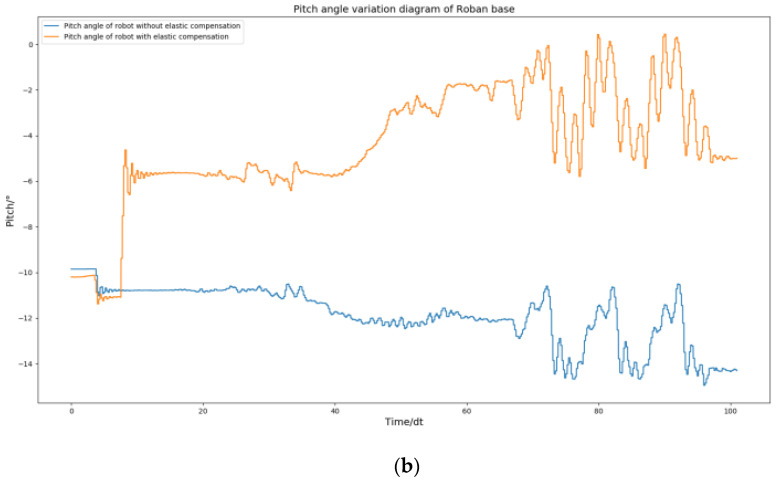
When Roban’s COM moves 5 cm, it completes three squats on a single leg. The comparison chart displays the roll angle and pitch angle with and without spring compensation. (**a**) Comparison diagram of roll angle change of robot with and without spring compensation. (**b**) Comparison diagram of pitch angle change of robot with and without spring compensation.

The effectiveness of spring compensation in correcting the error compensation of Roban can be observed from Figure 15. To further demonstrate its impact, we conducted another set of squat experiments where the COM shift was set at 4.3 cm. Similar to the previous experiment, the Roban robot performed a one-legged squat motion trajectory with position control. However, without spring compensation, the robot fell over during the experiment.

**Figure 16:**
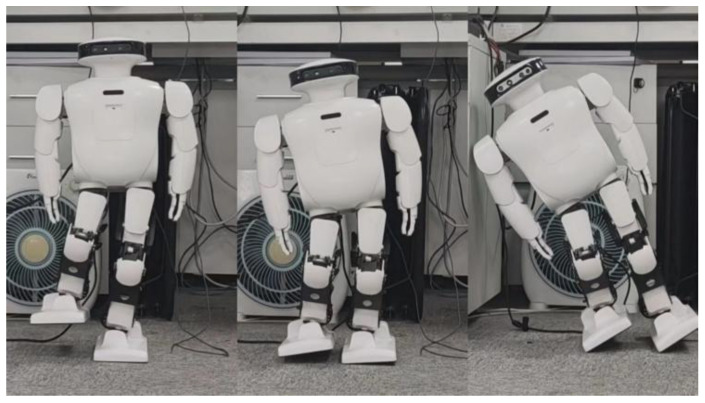
When Roban’s COM moves 4.3 cm, the robot fell while squatting.

**Figure 17:**
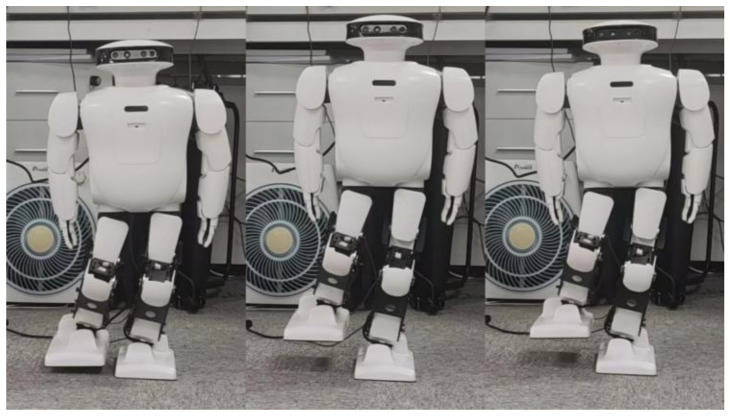
When Roban’s COM moves 4.3 cm, the robot completes the squat by means of spring compensation.

According to Figure 16, we can see that the robot has a roll. This means that when the robot raises and swings its legs, the hip will roll over due to gravity. When the roll angle was too high, the robot fell sideways, so we applied the spring compensation method to the robot executing this action trajectory. As a result, the robot was able to finish squatting as shown in Figure 17.

After obtaining the curve of roll angle and pitch angle under the compensation, we again compared the curve of roll angle and pitch angle under the no-spring compensation.

**Figure 18:**
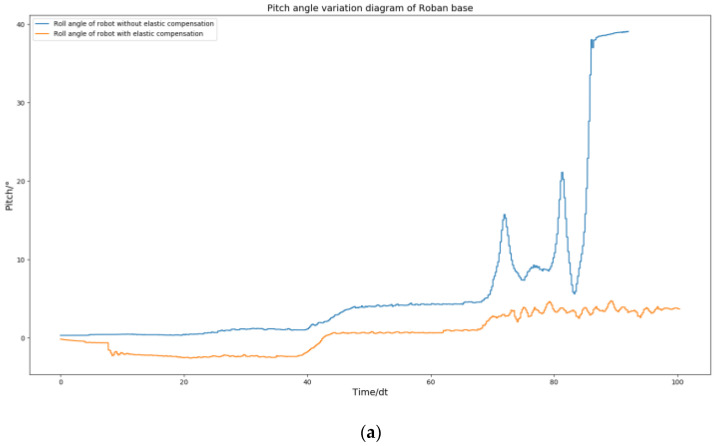

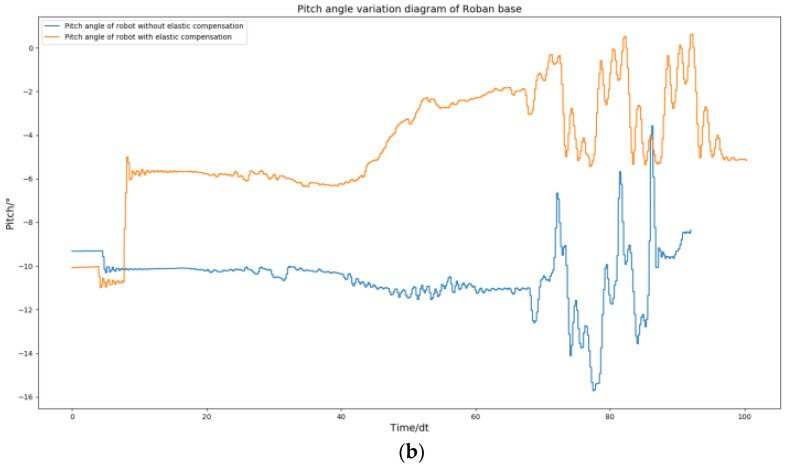
When Roban’s COM moves 4.3 cm, and completes three squats on a single leg. The comparison chart shows the difference in roll angle and pitch angle between the robot with and without spring compensation. (**a**) Comparison diagram of roll angle change of robot with and without spring compensation. (**b**) Comparison diagram of roll pitch change of robot with and without spring compensation.

The results shown in Figure 18 indicate that the robot lost its balance without spring compensation, while with spring compensation, it was able to move smoothly. To demonstrate the general applicability of this approach, several experiments were conducted in this study. The experiment included fast squat and deeper squat with the COM offset of 5 cm.

We conducted another experiment where the Roban robot performed a one-legged squat at double the speed. We executed this trajectory using both position control and compensation methods. Through this experiment, we aimed to investigate the impact of these two methods on the action.

**Figure 19:**
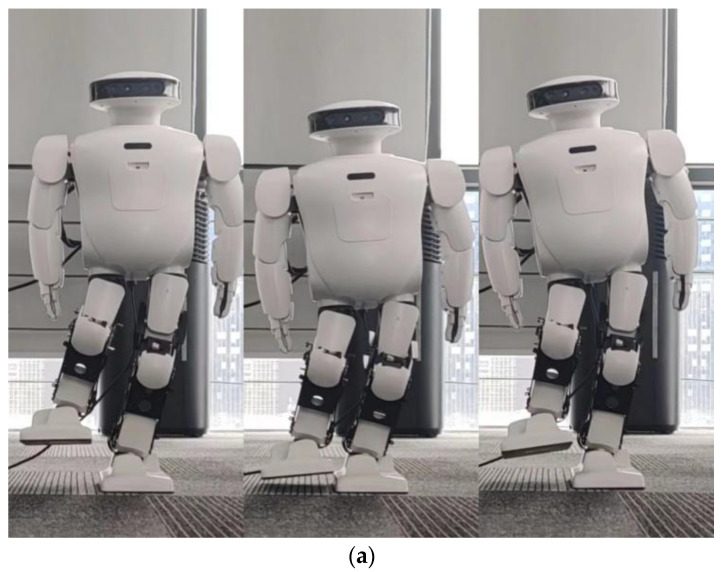

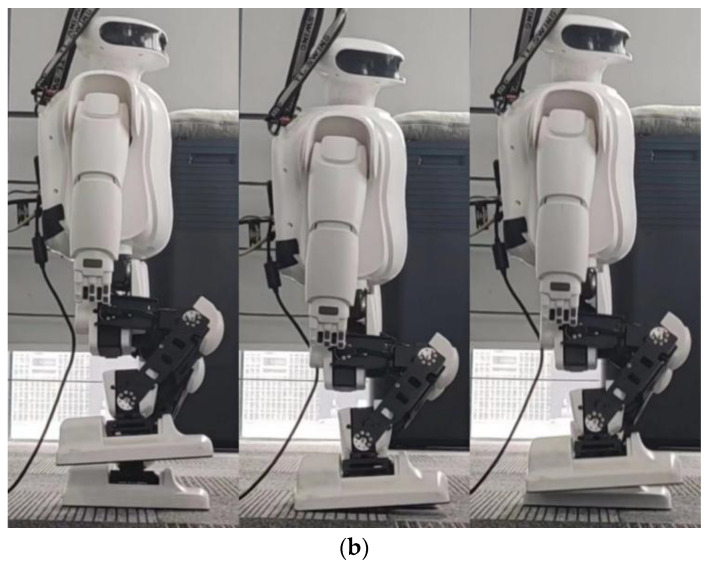
When Roban’s COM moves 5 cm, it completes three squats on a single leg at twice the speed. (**a**) A main view of the robot squatting process at twice the speed. (**b**) A side view of the robot squatting process at twice the speed.

From Figure 19, we can see that there are large errors in the process of robot squatting quickly. This shows that when the robot squats quickly, the hip joint will bear more variable load. We used spring compensation methods to compensate the robot that executes this motion trajectory.

**Figure 20:**
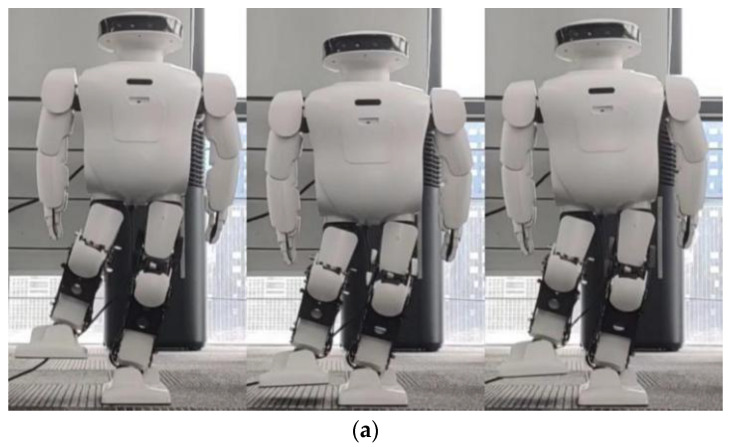

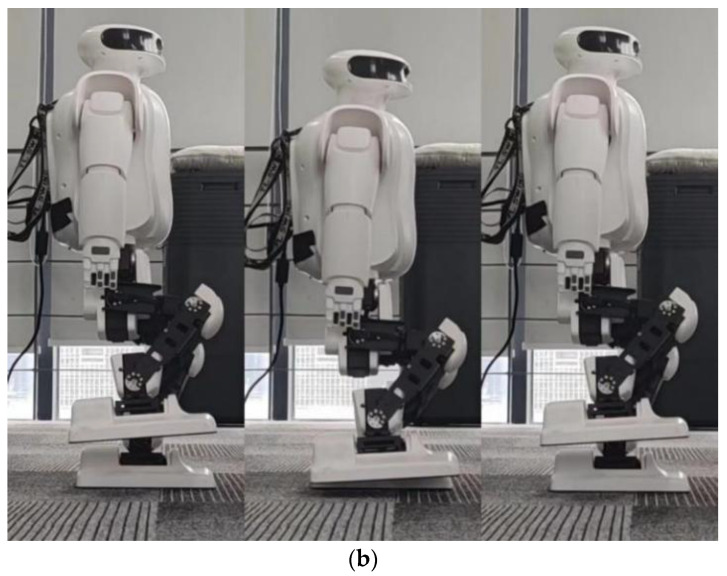
When Roban’s COM moves 5 cm, it completes three squats on a single leg at twice the speed using spring compensation. (**a**) A main view of the robot squatting process at twice the speed using spring compensation. (**b**) A side view of the robot squatting process at twice the speed using spring compensation.

Compared with the Figure 19 and Figure 20, the motion effect of the robot becomes better. We again note the roll and pitch angle curves between them.

**Figure 21:**
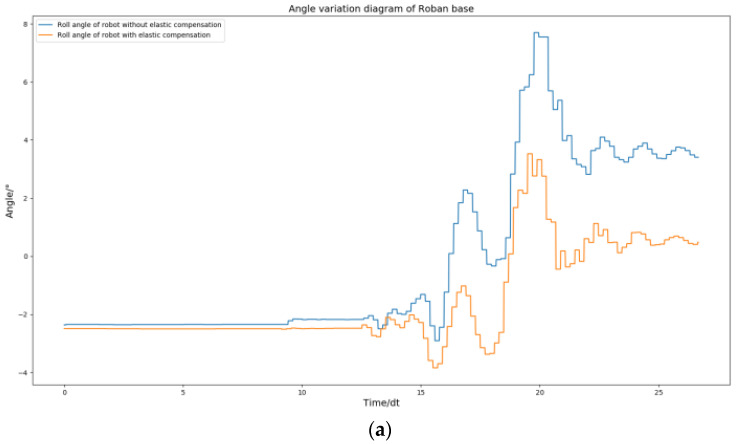

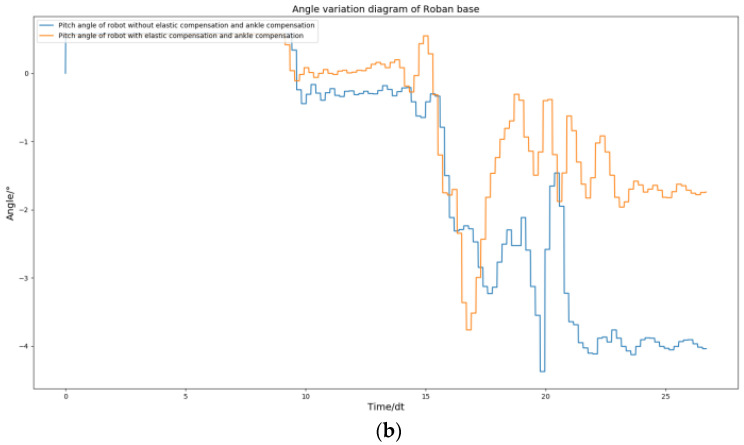
When Roban’s COM moves 5 cm, it competes three squats on a single leg at twice the speed. The comparison chart shows the roll angle and pitch angle with and without spring compensation. (**a**) Comparison diagram of roll angle change of robot with and without spring compensation. (**b**) Comparison diagram of pitch angle change of robot with and without spring compensation.

Figure 21 shows that by adopting the spring compensation, the robot can achieve a movement closer to the zero position. The spring compensation method improves the motion performance of the robot.

In order to further test the above conclusions, we will conduct another experiment that deepens the squatting depth of the robot when squatting at normal speed. We will increase the squatting range by 2 cm compared to the normal squatting range and complete this action without any compensation. The robot will use position control to perform the squatting action.

When the robot attempted to perform a deeper squat without compensation, it fell down while using only the position control mode. This indicates that when the robot raises its swinging leg, the hip joint experiences a roll due to gravity. When the roll angle is too large, the robot will fall to the side. This may cause damage to the robot. We try to adopt the spring compensation to do the same work.

**Figure 22:**
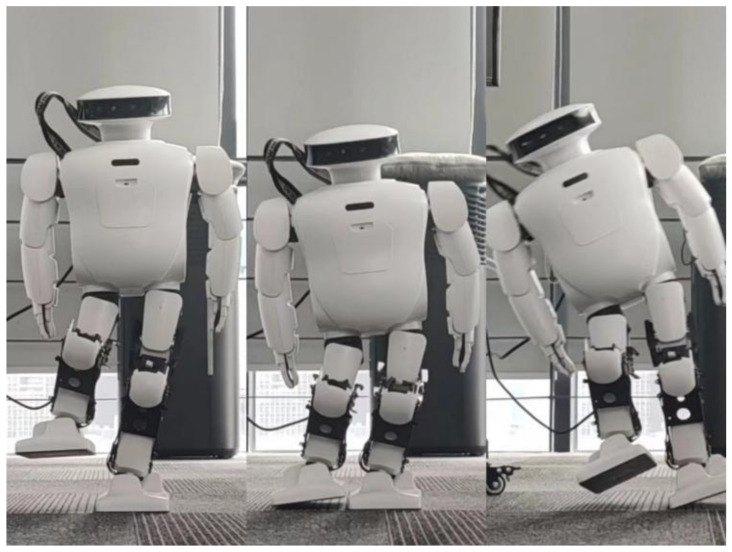
The robot fell down while performing a deeper squat.

**Figure 23:**
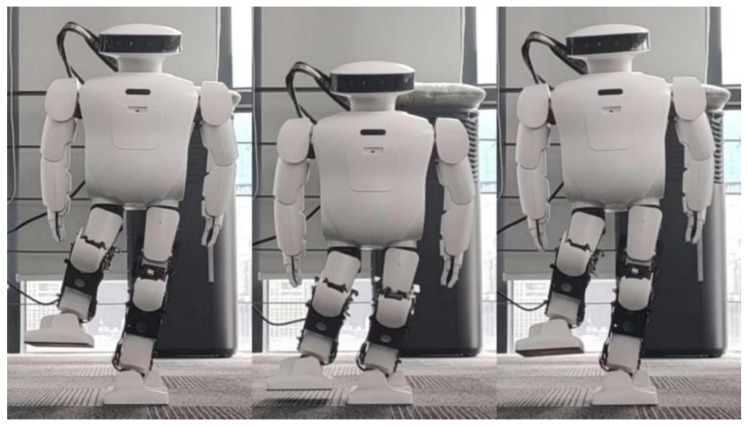
The robot performs a deeper squat with spring compensation.

The comparison between Figure 22 and Figure 23 shows the effect of this compensation method. The robot can complete squatting with increased squatting range in this compensation mode. After obtaining the roll angle and pitch angle curves under this compensation, we compared the roll angle and pitch angle curves under no spring compensation again.

**Figure 24:**
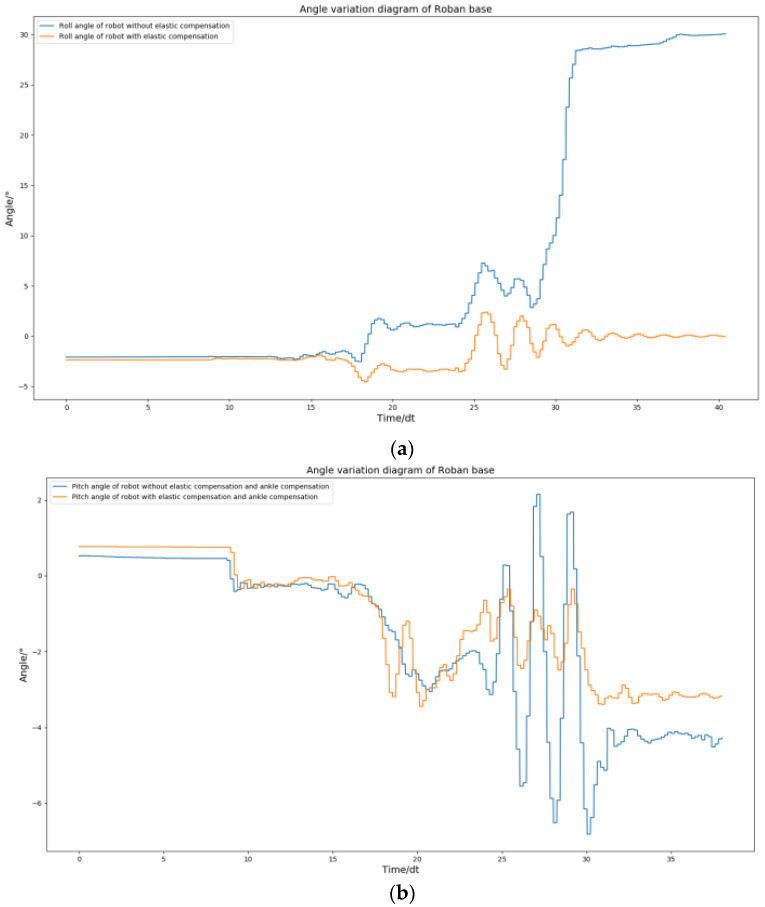
The robot performs a deeper squat. The comparison chart shows the roll angle and pitch angle with and without spring compensation. (**a**) Comparison diagram of roll angle change of robot with and without spring compensation. (**b**) Comparison diagram of roll angle change of robot with and without spring compensation.

Based on Figure 24, it is evident that the robot loses its balance without spring compensation. To address this, we enabled spring compensation, which allowed the robot to move smoothly. This confirms the effectiveness of the compensation method in dynamic actions.

### 5.4. Swing Experiment

**Figure 25:**
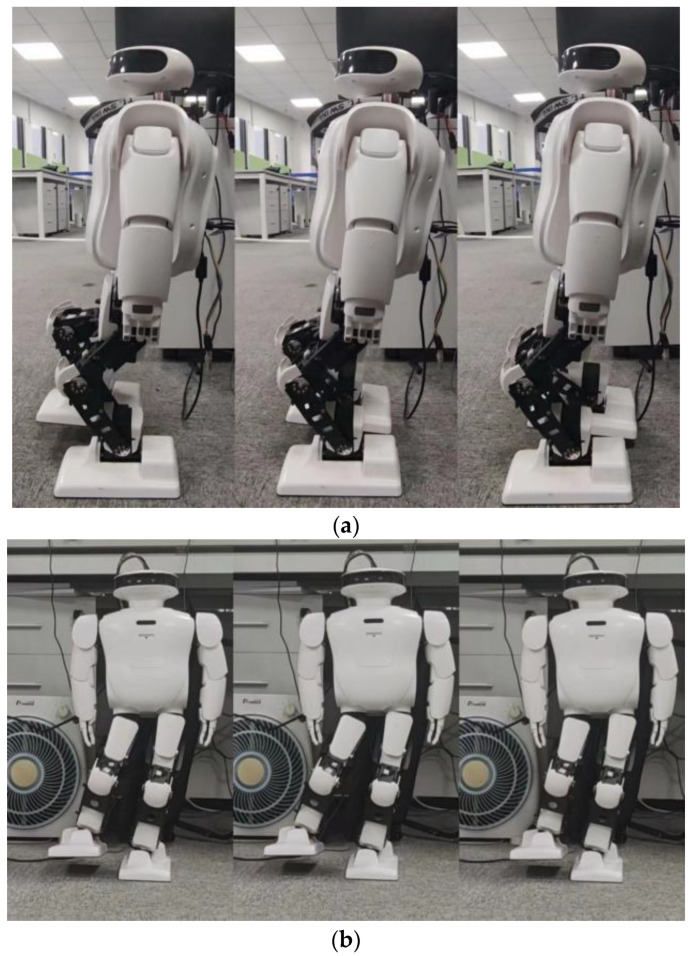
The Roban robot swings forward and sideways. (**a**) A side view of the robot swinging forward process. (**b**) A main view of the robot swinging sideways process.

The above experiment focused solely on squat actions, but to enhance the capabilities of low-cost robots, we must consider the movements humans perform in their daily lives, such as sideways walking and forward leg swings. These actions serve as the foundation of a robot’s walking pattern. To validate the spring compensation method, we conducted additional tests where the Roban robot performed side sway and front sway movements as shown in Figure 25.

An experiment was conducted on the side swing of a robot. The COM was moved 5 cm, and the experiment began with a position control test, similar to the previous chapter. The feedforward of joint torque was calculated through simulation, followed by an experiment involving spring compensation. During the experiment, data was collected on the roll and pitch angles of the Roban robot.

**Figure 26:**
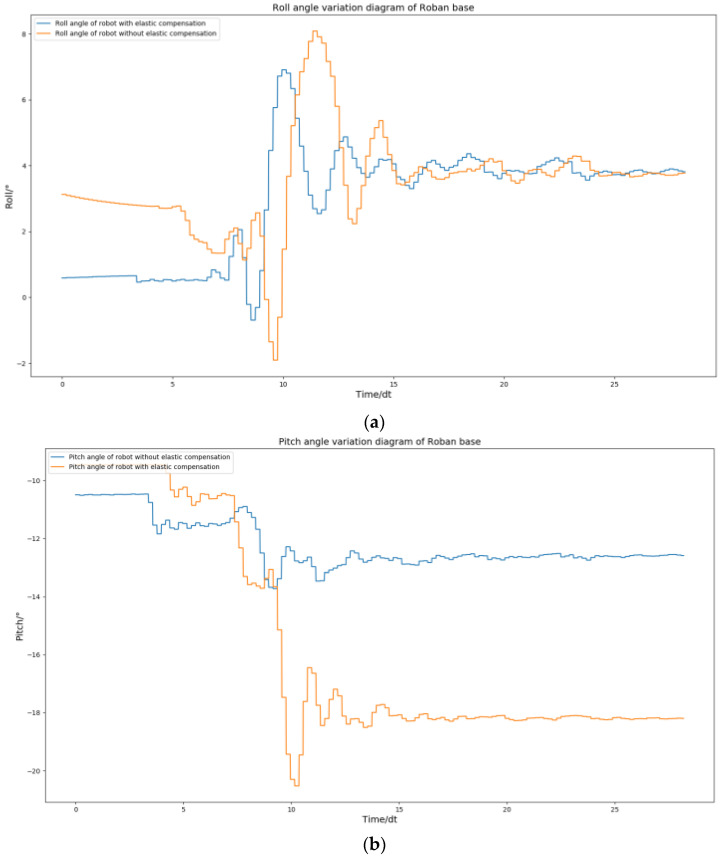
The Roban robot performs a side swing motion. We obtained the curve comparison diagram of roll angle and pitch angle of the robot with and without spring compensation in this process. (**a**) Comparison diagram of roll angle change of robot with and without spring Compensation. (**b**) Comparison diagram of pitch angle change of robot with and without spring compensation.

From the Figure 26, we can see that the spring compensation is not effective. When addressing such issues through spring compensation, the roll angle is typically unaffected, while the error in the pitch angle tends to increase. This is because when the robot performs dynamic actions, such as the side swing, the clearance error in the robot’s joint is amplified. Therefore, only spring compensation is used, and the robot still has errors when moving. In order to improve dynamic performance, we need to add clearance compensation on the basis of spring compensation.

Clearance compensation is compensated by a fixed angle. The direction of the angle is consistent with the direction of the angle after spring compensation. The specific formula of spring clearance compensation is as follows:

(23) $$q_{des}=\begin{array}{c} q+k\tau \end{array}+\{\begin{array}{c} \phi \;if\left(q+k\tau \right)>0\\ -\phi \;if\left(q+k\tau \right)<0 \end{array}$$

The ϕ is the clearance compensation angle. We set this angle to 8 degrees through the trial-and-error method mentioned in the previous section.

**Figure 27:**
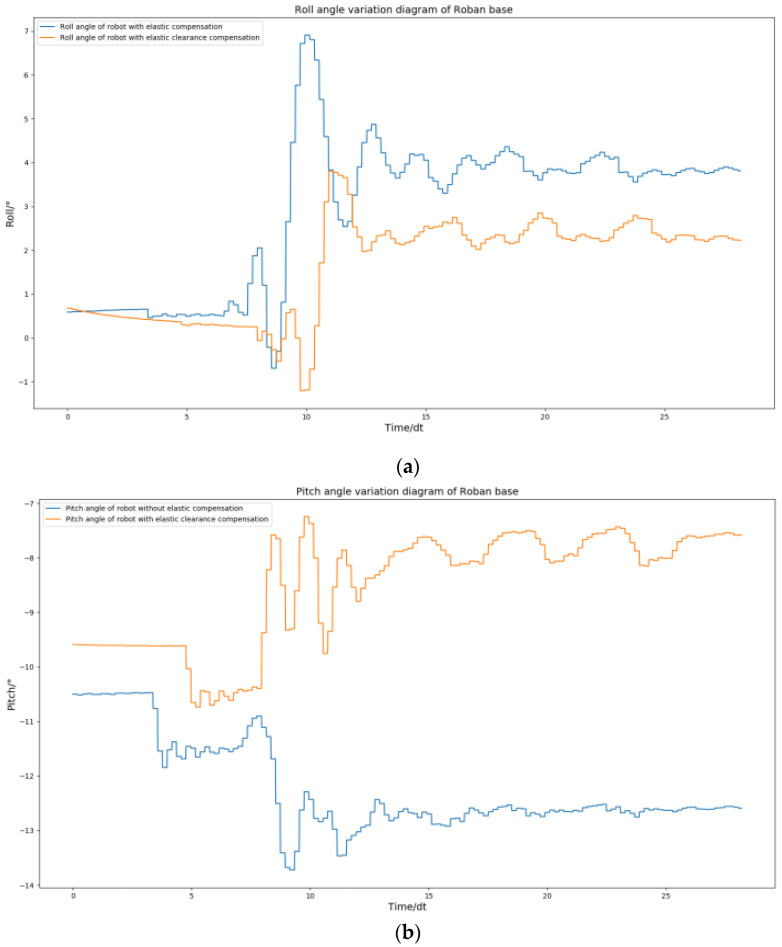
The Roban robot performs a side swing motion. We obtained the curve comparison diagram of roll angle and pitch angle of the robot with and without spring clearance compensation in this process. (**a**) Comparison diagram of roll angle change of robot with and without spring compensation. (**b**) Comparison diagram of pitch angle change of robot with and without spring compensation.

Next, we review our choices for the use of spring compensation. We decided to implement compensation on the key joint drivers, particularly for the NO. 3 steering gear due to significant error in the pitch direction. The remaining drivers retained their original compensation scheme. With this setup, we conducted a single-leg side swing test on the Roban robot and collected data on the roll and pitch angle curves during the action. In accordance with Figure 27, we can confirm the role of spring clearance compensation. The pitch direction error of the Roban robot can be further amplified with the implementation of spring compensation. However, when clearance compensation is introduced, the pitch direction error of the Roban robot becomes very low. At the same time, the roll angle error of the robot will become lower after the clearance compensation is adopted.

In order to verify the universality of spring clearance compensation, we then conducted the experiment of the robot’s single leg swinging back and forth. Here, we changed the side sway motion of the robot to back and front swinging. This kind of action will further test the stability of the robot in the pitch direction. We use position control to complete the action. Then, the action in the way of spring clearance compensation is completed. In this process, we collected the curves of roll angle and pitch angle.

**Figure 28:**
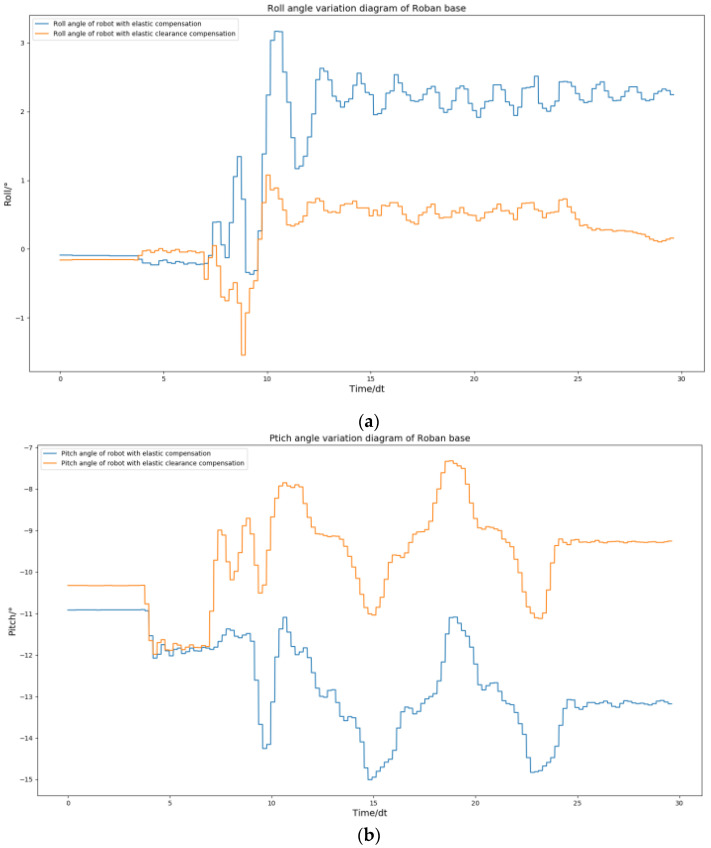
The Roban robot performs a forward swing motion. We obtained the curve comparison diagram of roll angle and pitch angle of the robot with and without spring clearance compensation in this process. (**a**) Comparison diagram of roll angle change of robot with and without spring compensation. (**b**) Comparison diagram of pitch angle change of robot with and without spring compensation.

As shown in Figure 28, the spring clearance compensation is still effective in dealing with the motion of the robot swinging its leg back and forth. The robot roll angle is finally able to return to a stable position. Similarly, its pitch angle can return to a stable position. This shows that the spring clearance compensation is effective in dealing with dynamic actions with forward or sideward motion.

## 6. Conclusions

We utilized LQR to control the robot’s COM and the whole-body-control method to compute the joint torque feedforward through simulation. We also presented the modeling method for spring compensation and how to obtain the spring coefficient. During the experiment, when we only used position control to execute the action, the Roban robot exhibited significant errors in the roll and pitch angles of its upper body.

The robot exhibited a very large wobble when performing the task. The robot was also unable to complete assigned tasks due to this problem. For squatting actions, we can directly use spring compensation to eliminate the above errors. However, when the robot took a forward or side swing-type action, the spring compensation was not effective in the direction of the pitch angle of the robot. Therefore, we changed the spring compensation to spring clearance compensation. This method can be used to compensate for the errors of such actions. The experimental results show that the spring compensation method is both effective and reasonable. To review the above experiments, we only conducted squat-type experiments. More practical walking experiments for robots have not yet been studied. At the same time, the torque generated by the whole-body-motion control has a large vibration, which will have a certain influence on the spring compensation. Moreover, the calculation of the joint torque of the robot by simulation will produce some errors. To add more walking experiments and eliminate the error of spring compensation will be the focus of our subsequent research.

## Figures and Tables

**Table 1:** Specific parameters of Roban robot.

DOF	22
Single leg DOF	6
Entire robot mass/kg	6.6
Entire robot height/cm	70

**Table 2:** Specific parameters of Roban robot’s steering gears.

Number	$q_{des}/{}^{\circ}$	q/°	τ/N·m	k
1	−0.00026	−0.23438	−0.00197	−118.74
2	0.6226	0.15625	−0.36474	−1.28
3	−37.9075	−36.6406	0.01779	−71.21
4	68.7407	71.6406	−1.86625	1.55
5	−30.8239	−28.9844	0.00371	−495.82
6	−0.6245	−1.40625	0.01724	45.36
7	0.00017	0.23438	0.00198	−118.29
8	−0.60888	−0.46874	−0.39568	0.35
9	−37.9126	−37.1875	−0.004	181.28
10	68.7484	71.4062	1.88342	−1.41
11	−30.8266	−29.375	0.02008	−722.91
12	0.60647	1.0156	−0.01592	25.70

**Table 3:** Specific parameters of Roban.

Number	k
2	−4
3	−650
8	20
9	−1200

## Data Availability

The data presented in this study are available on request from the corresponding author. The data are not publicly available due to only available to teams interested in collaboration.
